# Schlafen 11 Expression in Patients With Small Cell Lung Cancer and Its Association With Clinical Outcomes

**DOI:** 10.1111/1759-7714.15529

**Published:** 2025-01-14

**Authors:** Ken Masuda, Tatsuya Yoshida, Noriko Motoi, Yuki Shinno, Yuji Matsumoto, Yusuke Okuma, Yasushi Goto, Hidehito Horinouchi, Noboru Yamamoto, Shun‐ichi Watanabe, Tomoaki Hoshino, Yasushi Yatabe, Yuichiro Ohe

**Affiliations:** ^1^ Department of Thoracic Oncology National Cancer Center Hospital Tokyo Japan; ^2^ Department of Experimental Therapeutics National Cancer Center Hospital Tokyo Japan; ^3^ Department of Diagnostic Pathology National Cancer Center Hospital Tokyo Japan; ^4^ Division of Genome Biology National Cancer Center Research Institute Tokyo Japan; ^5^ Department of Pathology Saitama Cancer Center Saitama Japan; ^6^ Department of Thoracic Surgery National Cancer Center Hospital Tokyo Japan; ^7^ Division of Respirology, Neurology, and Rheumatology, Department of Medicine Kurume University School of Medicine Fukuoka Japan

**Keywords:** immunohistochemistry, lung cancer, Schlafen 11, small cell lung cancer

## Abstract

**Background:**

Schlafen 11 (SLFN‐11) has been identified as a sensitizer of tumor cells to DNA‐damaging agents. However, the relationship between SLFN‐11 expression and clinical outcomes in patients with small cell lung cancer (SCLC) remains unexplored. Thus, we aimed to evaluate the impact of SLFN‐11 expression on survival in patients with limited‐stage (LS) SCLC.

**Methods:**

We conducted a retrospective review of data from patients pathologically diagnosed with LS‐SCLC post‐surgery between January 2008 and December 2018. SLFN‐11 expression was assessed using immunohistochemistry in tissue microarrays and scored using a histology (H)‐score (range: 0–300).

**Results:**

Overall, 86 patients were included in the analysis with a median H‐score of 43 for SLFN‐11 expression. Among the patients, 44 had high SLFN‐11 expression (provisionally defined as H‐score ≥ 43). No significant differences in clinical profiles were observed between the two groups (high and low SLFN expression). The median survival durations were not reached (NR; 95% confidence interval [CI]: 65.1 months to NR) and 33.5 months (95% CI: 24.2 months to NR) for patients with high and low SLFN‐11 expression, respectively (hazard ratio [HR]: 0.40, 95% CI: 0.19–0.81; *p* = 0.012). Among patients who relapsed post‐surgery (*n* = 21), the median survival durations were 22.0 (95% CI: 7.6–44.9 months) and 8.1 (95% CI: 1.8–24.6 months) months in patients with high and low SLFN‐11 expression, respectively (HR: 0.22, 95% CI: 0.06–0.84; *p* = 0.026).

**Conclusions:**

High SLFN‐11 expression is associated with relatively longer survival in patients with LS‐SCLC in both those undergoing surgery and those who have relapsed.

## Graphical abstract

We conducted a retrospective review of patients who underwent surgery for LS‐SCLC and were pathologically diagnosed with the disease. Using immunohistochemistry on tissue microarrays, we evaluated the impact of SLFN‐11 expression on survival. We found that patients with high SLFN‐11 expression had a more prolonged median survival, suggesting that SLFN‐11 may serve as a valuable indicator of cytotoxic chemotherapy efficacy.

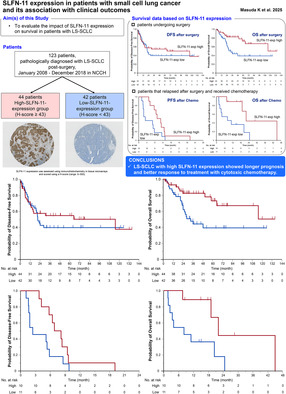

**Abbreviations**
DFS: disease‐free survival
ECOG‐PS: Eastern Cooperative Oncology Group performance status
IHC: immunohistochemistry
LS‐SCLC: limited‐stage small cell lung cancer
ORR: objective response rate
OS: overall survival
PFS: progression‐free survival
SLFN‐11: Schlafen 11

## 1 Background

Small cell lung cancer (SCLC) accounts for 14%–15% of all lung cancer diagnoses [1]. SCLC is considered an aggressive and smoking‐related malignancy, and its 5‐year survival rate ranges from 20% to 25% for limited‐stage (LS) disease; however, the rate drops to approximately 2% for extensive‐stage (ES) disease [2, 3]. Initially, SCLC is highly responsive to platinum‐based chemotherapy or radiotherapy, leading to significant tumor reduction; however, SCLC often relapses and develops resistance to chemotherapy. Although platinum‐based chemotherapy has been used as the standard treatment for the past 30 years, there has been a surge in the identification of various genetic abnormalities and signaling pathways associated with SCLC in recent years [3, 4]. Combining immune checkpoint blockade with platinum‐based chemotherapy has significantly increased survival in patients with ES‐SCLC [5, 6]. However, the contribution of immune checkpoint inhibitors (ICIs) to survival in SCLC has not been elucidated. Therefore, cytotoxic anticancer agents remain the mainstay of SCLC treatment.

SCLC is a transcriptionally active disease characterized by common loss‐of‐function genomic alterations in the tumor suppressor genes *p53* and RB transcriptional corepressor 1 (*RB1*), which creates further genomic instability by preventing arrest of the cell cycle required for performing DNA repair [7, 8, 9, 10]. The abrogation of the G1‐S cell cycle checkpoint, associated with the loss of p53 and RB1, results in an increased reliance on G2‐M cell cycle checkpoints to ensure genome stability and appropriate chromosomal segregation [4]. Recent studies have highlighted the potential of targeting the DNA damage response (DDR) pathway as a promising therapeutic strategy for SCLC [11, 12, 13, 14, 15, 16]. Although emerging candidate biomarkers may help identify subsets of SCLC, which are vulnerable to specific DDR inhibitors, robust biomarkers comparable to the *BRCA* gene in ovarian or breast cancer have not yet been identified [17, 18, 19, 20].

Schlafen 11 (SLFN‐11) was identified via bioinformatics analyses of cancer cell databases as a dominant determinant of responses to widely used anticancer drugs and is expected to represent a biomarker that can predict the efficacy of cytotoxic anticancer drugs and DDR inhibitors [21, 22]. The utility of SLFN‐11 as a predictor of therapeutic response to DNA cross‐linking agents and poly (ADP‐ribose) polymerase (PARP) in SCLC has been reported in vivo and in vitro [23, 24]. Additionally, the utility of SLFN‐11 as a marker has been examined in ES‐SCLC in a randomized controlled phase II trial comparing the combination of veliparib and temozolomide to veliparib and a placebo [25]. In this clinical trial, significantly prolonged progression‐free survival (PFS; 5.7 vs. 3.6 months) and overall survival (OS; 12.2 vs. 7.5 months) were observed in patients with SLFN‐11‐positive tumors treated with veliparib and temozolomide. However, no studies have examined the role of SLFN‐11 in the prognosis and prediction of response to treatments such as platinum‐based chemotherapy following recurrence in LS‐SCLC. Therefore, we aimed to investigate the association between SLFN‐11 expression and survival of patients with LS‐SCLC undergoing surgery. We hypothesized that high SLFN‐11 expression is associated with relatively longer survival in these patients owing to the differential effects of platinum‐based chemotherapy post‐surgery.

## 2 Methods

### 2.1 Study Design and Subjects

This study was performed in accordance with the ethical standards of the Declaration of Helsinki and the ethical guidelines for epidemiological research presented by the Ministry of Health, Labour, and Welfare in Japan. The study protocol was reviewed and approved by the Ethics Committee of the National Cancer Center Hospital (2019‐123). Informed consent from the patients was waived.

This retrospective study included patients who were pathologically diagnosed with LS‐SCLC post‐surgery between January 2008 and December 2018. Patients who had a sufficient number of tumor tissues for evaluating SLFN‐11 expression via immunohistochemistry (IHC) in tissue microarrays were selected. We reviewed the medical records and extracted the following patient characteristics: age, sex, Eastern Cooperative Oncology Group performance status (ECOG‐PS), histology (H), smoking history, disease status, details of treatment, and survival. The subjects selected were assigned to two groups according to the SLFN‐11 expression level (high and low). The survival and efficacy of chemotherapy after surgery in the two groups was assessed by evaluating disease‐free survival (DFS), PFS, and OS.

### 2.2 Analysis of SLFN‐11 Expression

**FIGURE 1:**
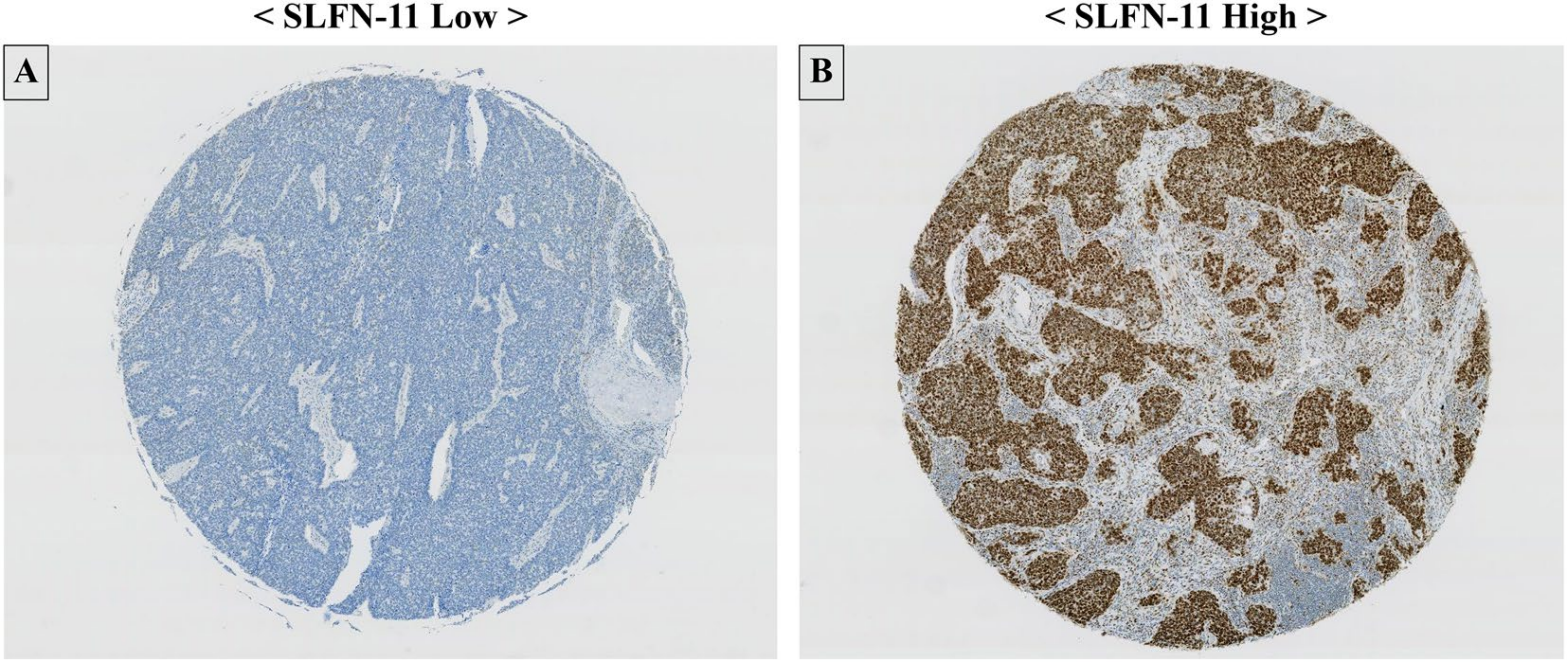
Pathological images of small cell lung cancer samples subjected to immunohistochemistry. SLFN‐11, Schlafen 11; A, low SLFN‐11 expression; B, high SLFN‐11 expression.

Formalin‐fixed paraffin‐embedded specimens obtained after surgery were subjected to immunohistochemical staining using anti‐SLFN‐11 mouse monoclonal antibodies (clone D2, sc‐515 071; Santa Cruz Biotechnology, Dallas, TX, USA) at a 1:50 dilution (Figure 1) [26, 27, 28]. All stained slides were digitalized using a NanoZoomer 2.0‐HT Whole Slide Imager (Hamamatsu Photonics, Hamamatsu, Japan) at 40× resolution. The SLFN expression level was evaluated based on the H‐score in the annotated tumoral area. Tumoral area was manually annotated by two researchers (KM and NM, including one certified pathologist). The H‐score was calculated by multiplication of two parameters: proportion of positive cells (1%–100%) and intensity of labeling (1 = weak, 2 = moderate, 3 = strong; range, 0–300) using the Halo platform (Indica Labs, Albuquerque, NM, USA) and confirmed by a pathologist (NM). Since there was no consensus on the SLFN‐11 expression cutoff value, we decided to set a median H‐score of 43 as the SLFN‐11 expression cutoff value. Study subjects were assigned to the two groups based on this SLFN‐11 expression cut‐off.

### 2.3 Statistical Analysis

Differences between groups were analyzed using Fisher's exact test for categorical variables. The objective response rate (ORR) in patients with target lesions was evaluated based on the Response Evaluation Criteria in Solid Tumors version 1.1 and assessment via computed tomography every 6 to 8 weeks after the start of chemotherapy following recurrence. DFS was defined as the time from surgery to first locoregional or distant recurrence or to death. PFS was defined as the time from chemotherapy after recurrence to progression or to death from any cause. OS was defined as the time from surgery or initial day of chemotherapy to death from any cause. DFS, PFS, and OS were estimated using the Kaplan–Meier method and compared using the log‐rank test. All statistical analyses were performed using JMP version 14.0 (SAS Institute, Cary, NC, USA). All *p* values were two‐sided, and *p* < 0.05 was considered significant.

## 3 Results

### 3.1 Patient Characteristics and SLFN‐11 Expression

In total, 86 patients were included in this study (Figure S1). Patient characteristics are summarized in Table 1. The median age was 69 years (range, 46–88 years), and 73 patients (84.9%) were male. In total, 81 patients (94.2%) had an ECOG‐PS of 0 or 1, and 53 patients (61.6%) received adjuvant chemotherapy after surgery. The median H‐score of SLFN‐11 expression was 43 (range, 1–292), and high SLFN‐11 expression was defined based on an H‐score of ≥ 43. Characteristics of patients in the high‐ (H‐score ≥ 43) and low‐ (H‐score < 43) SLFN‐11‐expression groups are summarized in Table 1. No significant differences in clinical profiles were observed between the two groups. Table 2 shows the characteristics of the 21 patients that had relapsed and received chemotherapy; among these, 10 (47.6%) showed high SLFN‐11 expression. No significant differences in clinical profiles were observed between the two groups.

**TABLE 1:** Characteristics of the patients in the high (H‐score ≥ 43) and low (H‐score < 43) SLFN‐11 expression groups.

	All patients, no. (%)	SLFN‐11, high group, no. (%)	SLFN‐11, low group, no. (%)	*p*
Total *N*	86	44	42	
Median age, years (range)	69 (46–88)	69 (57–83)	69 (46–88)	
Sex				0.06
Female	13 (15.1)	8 (18.2)	5 (11.9)	
Male	73 (84.9)	36 (81.8)	37 (88.1)	
ECOG PS				0.875
0, 1	81 (94.2)	42 (95.4)	39 (72.9)	
≥ 2	5 (5.8)	2 (4.6)	3 (7.1)	
Smoking history				1.000
Never smoker	2 (2.3)	1 (2.3)	1 (2.4)	
Smoker	84 (97.7)	43 (97.7)	41 (97.6)	
Histologic classification				0.294
SCLC	68 (79.1)	37 (84.1)	31 (73.8)	
Combined SCLC	18 (20.9)	7 (15.9)	11 (26.2)	
Disease status				0.452
Stage I	53 (59.3)	27 (61.4)	24 (57.1)	
Stage II	24 (27.9)	13 (29.6)	11 (26.2)	
Stage III	8 (9.3)	2 (4.6)	6 (14.3)	
Stage IV	3 (3.5)	2 (4.6)	1 (2.4)	
Adjuvant chemotherapy				0.825
Performed	53 (61.6)	28 (63.6)	25 (59.5)	
Non‐performed	33 (38.4)	16 (36.4)	17 (40.5)	

Abbreviations: ECOG PS, Eastern Cooperative Oncology Group performance status; SCLC, small cell lung cancer; SLFN‐11, Schlafen 11.

**TABLE 2:** Characteristics of the recurrent patients who received chemotherapy in the high (H‐score ≥ 43) and low SLFN‐11 (H‐score < 43) expression groups.

	All patients, no. (%)	SLFN‐11, high group, no. (%)	SLFN‐11, low group, no. (%)	*p*
Total *N*	21	10	11	
Median age, years (range)	69 (57–79)	69 (57–79)	69 (57–79)	
Sex				1.000
Female	2 (9.5)	1 (10.0)	1 (9.1)	
Male	19 (90.5)	9 (90.0)	10 (90.9)	
ECOG PS				0.621
0, 1	20 (85.2)	10 (100)	10 (90.9)	
≥ 2	1 (4.8)	0 (0)	1 (9.1)	
Smoking history				1.000
Never smoker	0 (0)	0 (0)	0 (0)	
Smoker	21 (100)	10 (100)	11 (100)	
Histologic classification				0.311
SCLC	16 (76.2)	9 (90.0)	7 (63.6)	
Combined SCLC	5 (23.8)	1 (10.0)	4 (36.4)	
Disease status				0.856
Stage I	8 (38.1)	4 (40.0)	4 (36.4)	
Stage II	8 (38.1)	3 (30.0)	5 (45.5)	
Stage III	3 (14.3)	2 (20.0)	1 (9.1)	
Stage IV	2 (9.5)	1 (10.0)	1 (9.1)	
Chemotherapy regimen				0.705
Platinum‐based	15 (71.4)	8 (80.0)	7 (63.6)	
AMR	3 (14.3)	1 (10.0)	2 (18.2)	
Others	3 (14.3)	1 (10.0)	2 (18.2)	

Abbreviations: AMR, amrubicin; ECOG PS, Eastern Cooperative Oncology Group performance status; SCLC, small cell lung cancer; SLFN‐11, Schlafen 11.

### 3.2 Survival Data Based on SLFN‐11 Expression

**FIGURE 2:**
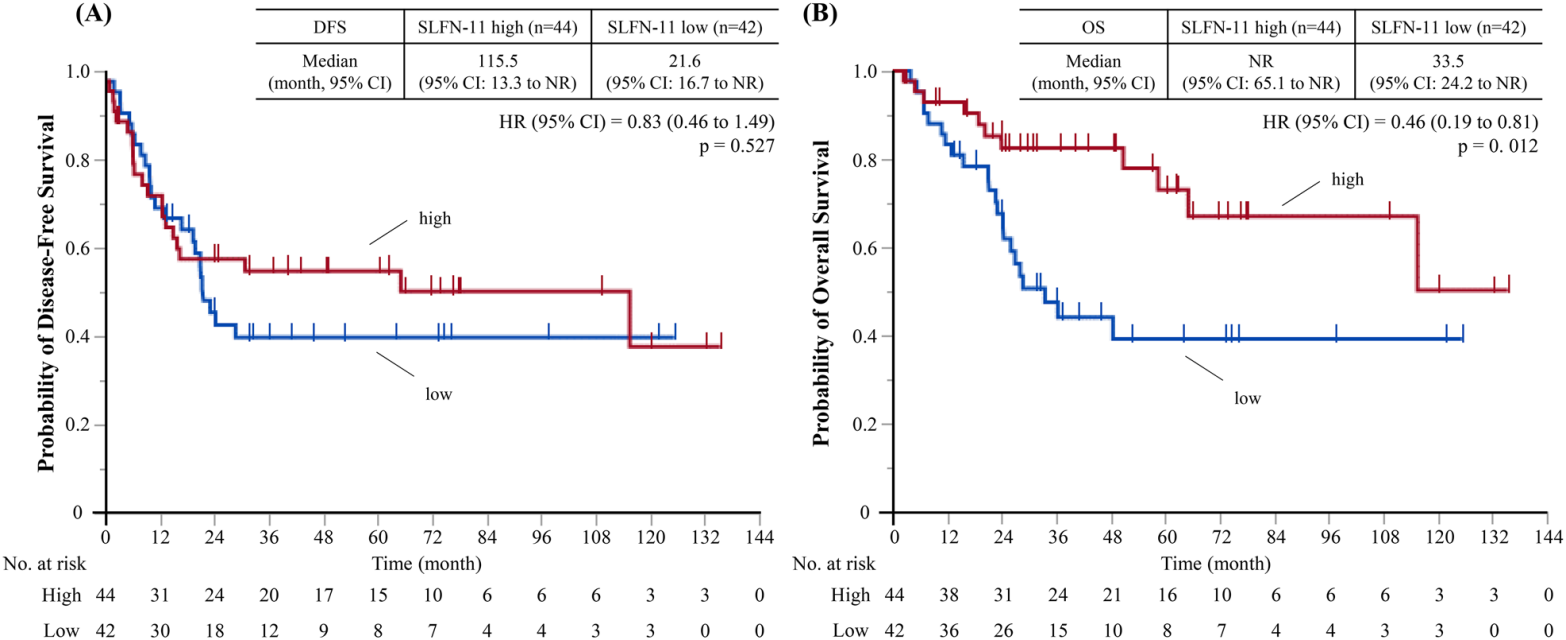
Kaplan–Meier curves for (A) DFS and (B) OS in patients with limited‐stage small cell lung cancer undergoing surgery. DFS, disease‐free survival; OS, overall survival.

The median follow‐up period for the 86 patients was 49.1 months (95% confidence interval [CI], 37.3–66.1 months). The median DFS was 28.7 months (95% CI, 16.7 months to not reached [NR]), and the median OS was 115.5 months (95% CI, 48.5 months to NR). Figure 2 shows Kaplan–Meier curves for DFS and OS according to the SLFN‐11 expression level in patients with LS‐SCLC undergoing surgery. The median DFSs were 111.5 months (95% CI, 13.3 months to NR) and 21.6 months (95% CI, 16.7 months to NR) in the patients with high and low SLFN‐11 expression values, respectively (hazard ratio [HR], 0.83; 95% CI, 0.46–1.49; *p* = 0.527). The median OSs were NR (95% CI, 65.1 months to NR) and 33.5 months (95% CI, 24.2 months to NR) in patients with high and low SLFN‐11 expression values, respectively (HR, 0.40; 95% CI, 0.19–0.81; *p* = 0.012).

### 3.3 Efficacy of Chemotherapy Based on SLFN‐11 Expression

**FIGURE 3:**
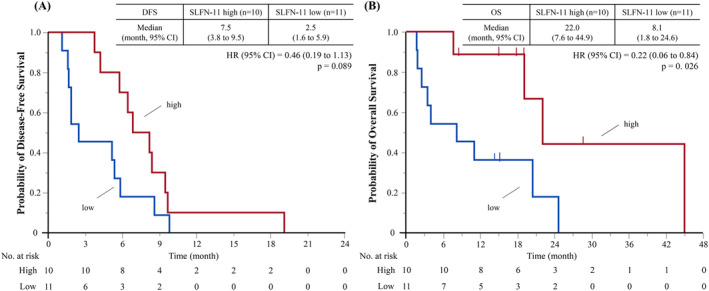
Kaplan–Meier curves for (A) DFS and (B) OS in patients with limited‐stage small cell lung cancer who relapsed after surgery and received chemotherapy. DFS, disease‐free survival; OS, overall survival.

In patients that relapsed after surgery and received chemotherapy, the ORR was 80.0% (95% CI, 49.0–94.3%) in the high‐SLFN‐11‐expression group (*n* = 10) and 27.2% (95% CI, 9.7–56.6%) in the low‐SLFN‐11‐expression group (*n* = 11). Figure 3 shows the Kaplan–Meier curves for PFS and OS based on the SLFN‐11 expression level in patients with LS‐SCLC who relapsed after surgery and received chemotherapy. The median PFSs were 7.5 months (95% CI, 3.8–9.5 months) and 2.5 months (95% CI, 1.6–5.9 months) in patients with high and low SLFN‐11 expression, respectively (HR, 0.46; 95% CI, 0.19–1.13; *p* = 0.089). The median OSs were 22.0 months (95% CI: 7.6–44.9 months) and 8.1 months (95% CI: 1.8–24.6 months) in patients with high and low SLFN‐11 expression, respectively (HR: 0.22; 95% CI: 0.06–0.84; *p* = 0.026). Figure S2 shows the Kaplan–Meier curves for DFS and OS based on the SLFN‐11 expression level in patients with LS‐SCLC who received adjuvant chemotherapy after surgery. The median DFSs were 115.5 months (95% CI, 16.4–NR months) and NR months (95% CI, 21.6–NR months) in patients with high and low SLFN‐11 expression, respectively (HR, 0.82; 95% CI, 0.33–2.03; *p* = 0.668). The median OSs were NR months (95% CI: 115.5–NR months) and NR months (95% CI: 28.1–NR months) in patients with high and low SLFN‐11 expression, respectively (HR: 0.28; 95% CI: 0.09–0.91; *p* = 0.026).

## 4 Discussion

We evaluated the expression of SLFN‐11 and its prognostic impact using surgical specimens previously obtained from patients with LS‐SCLC. To achieve a more accurate assessment of SLFN‐11 expression, we utilized surgical specimens from patients with LS‐SCLC rather than biopsy specimens from those with ES‐SCLC. Survival analyses of 86 patients with LS‐SCLC demonstrated favorable DFS and OS outcomes, and among those with high SLFN‐11 expression, patients who experienced postoperative recurrence achieved notably high response rates and improved PFS with chemotherapy. These findings strongly suggest that SLFN‐11 may serve as a predictive biomarker for the therapeutic efficacy of cytotoxic anticancer agents in SCLC.

SLFN‐11 sensitizes cells to a broad range of anti‐cancer drugs including platinum derivatives, topoisomerase inhibitors, DNA synthesis inhibitors, and PARP inhibitors. SLFN‐11 irreversibly blocks replication in cells under replication stress, which explains why SLFN‐11‐positive cells are killed more efficiently by DNA‐targeting drugs than SLFN‐11‐negative cells [26]. In addition, a recent study suggested an association between SLFN‐11 and the immune microenvironment. Zhou et al. reported that SLFN‐11 serves as a critical regulator of microenvironmental immune properties and an effective predictive biomarker of ICI response in hepatocellular carcinoma [29]. In clinical research, two randomized trials (IMpower133 trial and CASPIAN trial) examined the addition of ICIs to platinum‐doublet therapy in the first‐line setting of ES‐SCLC. The findings suggest that the combination of platinum‐doublet therapy and either atezolizumab or durvalumab regimens are becoming more widely used to treat ES‐SCLC [5, 6]. Cytotoxic anticancer agents represent the standard treatment for SCLC, and ICIs have been widely used. In addition to these factors, the fact that SLFN‐11 is highly expressed in SCLC suggests the importance of studying the relationship between SLFN‐11 expression and prognosis in SCLC in a clinical setting based on the results of in vivo and in vitro studies. This may have important implications for the treatment of SCLC in the future. In a recently conducted randomized Phase II trial, the efficacy and safety of atezolizumab plus talazoparib were reported in patients with ES‐SCLC who exhibited SLFN‐11 expression (H‐score ≥ 1) [30]. These findings highlight the potential of SLFN11‐guided treatment strategies to optimize therapeutic outcomes in SCLC, though further studies are needed to refine biomarker thresholds and there is a potential to add SCLC subtypes or Delta‐like ligand 3 as well to improve this potential marker to be synergistic to other biomarkers. In our study, only one patient received ICI therapy during postoperative recurrence. Since this patient exhibited low SLFN‐11 expression, it is unlikely that their case significantly impacted the response rates or survival analyses in the SLFN‐11 high‐expression group.

Few studies have focused on the association between the SLFN‐11 expression level and the efficacy of chemotherapy in patients with solid tumors. In patients with gastric and bladder cancer, high SLFN‐11 expression is a predictor of efficacy of platinum‐based chemotherapy [31, 32]. In a study on gastric cancer, 169 patients with advanced gastric cancer were analyzed for SLFN‐11 expression based on IHC. In patients who did not receive platinum‐based therapy (*N* = 121), there was no apparent difference in 5‐year survival between the high and low SLFN‐11 expression groups (*p* = 0.4484). However, the 5‐year survival rates for patients treated with platinum‐based chemotherapy (oxaliplatin or cisplatin; *N* = 48) were 64% and 0% for the high and low SLFN‐11 expression groups, respectively (*p* = 0.0009). In a study on bladder cancer, SLFN‐11 expression was determined via IHC in 120 patients with perioperative bladder cancer who had undergone surgery. Patients were assigned to two groups: those who received adjuvant or neoadjuvant chemotherapy in the perioperative period (*N* = 50) and those who did not (*N* = 70). The relationship between SLFN‐11 expression and survival was examined in each group. Patients who did not receive chemotherapy had a better prognosis in those who were SLFN‐11‐negative (*p* = 0.034), while those who received chemotherapy had a relatively better prognosis than those who were SLFN‐11‐positive (*p* = 0.012). In our study, postoperative patients with relatively higher SLFN‐11 expression had a better prognosis and response to treatment with platinum‐based chemotherapy, which gives validity to our results compared with those of previous studies.

This study had several limitations. First, because our study was a single‐center retrospective study rather than an interventional study, the patient population was small and heterogeneous. There may have been unmeasured confounders; however, we believe that the validity obtained in this study, especially the ORR, is reliable. Regarding ORR, previous randomized controlled trials have reported an ORR of 40%–70% [33, 34, 35, 36, 37]. However, the ORR of 27.2% for patients with low SLFN‐11 expression was relatively low. In patients with low SLFN‐11 expression, the H‐score of SLFN‐11 in the five patients who exhibited progression after chemotherapy (response decision: PD) was in the range of 7–10, which involved a low‐SLFN‐expressing population. This may have been due to the small number of patients; however, it suggests that patients with low expression of SLFN‐11 may not respond well to chemotherapy. Similar studies should be conducted to confirm our conclusions. Second, this study did not examine the relationship between SLFN‐11 expression and response to the combination of ICIs and platinum‐based chemotherapy, which is currently the standard of care for ES‐SCLC. SLFN‐11 expression should be examined using surgical specimens rather than biopsy specimens to more accurately examine SLFN‐11 expression. However, these therapies have been in use for only approximately 3 years, and the number of operable cases of SCLC itself is small; thus, we believe that the specimens are not ready for examination. Third, it remains unclear whether the cutoff value of SLFN‐11 expression in this study is relevant; SLFN‐11 expression was calculated based on the H‐score and the median was selected as the cutoff value. Although this cutoff is reasonable in the perspective of previous studies [27], the validity of the cutoff needs to be verified in large‐scale trials such as randomized controlled trials.

## 5 Conclusions

We used IHC to stain for SLFN‐11 to evaluate its role in prognosis post‐surgery and response to chemotherapy following recurrence; patients with LS‐SCLC with high SLFN‐11 expression showed longer prognosis and better response to treatment with cytotoxic chemotherapy. Future evaluations using biopsy specimens should be conducted to assess the role of SLFN‐11 in ES‐SCLC, particularly focusing on the efficacy of treatments involving ICIs and drugs related to DNA damage repair.

## Author Contributions

**Ken Masuda:** writing – original draft, writing – review and editing, data curation, conceptualization, formal analysis, visualization, investigation. **Tatsuya Yoshida:** writing – review and editing, methodology, supervision, resources, project administration. **Noriko Motoi:** writing – review and editing, methodology, software formal analysis, validation, visualization. **Yuki Shinno:** writing – review and editing. **Yuji Matsumoto:** writing – review and editing. **Yusuke Okuma:** writing – review and editing. **Yasushi Goto:** writing – review and editing. **Hidehito Horinouchi:** writing – review and editing. **Noboru Yamamoto:** writing – review and editing. **Shun‐ichi Watanabe:** writing – review and editing. **Tomoaki Hoshino:** writing – review and editing. **Yasushi Yatabe:** writing – review and editing. **Yuichiro Ohe:** writing – review and editing, supervision, project administration.

## Ethics Statement

This study was performed in accordance with the ethical standards of the Declaration of Helsinki and the ethical guidelines for epidemiological research presented by the Ministry of Health, Labour, and Welfare in Japan. The study protocol was reviewed and approved by the Ethics Committee of the National Cancer Center Hospital (2019–123). Informed consent from the patients was waived.

## Consent

The authors have nothing to report.

## Conflicts of Interest

K.M. reports receiving personal fees from Ono Pharmaceutical Co. Ltd., AstraZeneca, Chugai, and Bristol‐Myers Squibb, outside of the submitted work. T.Y. reports receiving grants and personal fees from Amgen, AstraZeneca, Ono, Merck Sharp & Dohme, Novartis, Chugai, and Bristol‐Myers Squibb; grants from Takeda, Daiichi Sankyo, and AbbVie; and personal fees from Taiho, Eli Lilly, Roche, and ArcherDX outside of the submitted work. Y.S. reports receiving personal fees from Bristol‐Myers Squibb, Chugai, AstraZeneca, and Eli Lilly; grants and personal fees from Ono; and grants from Janssen and Japan Clinical Research Operations K.K. outside of the submitted work. Y.M. reports receiving grants from the National Cancer Center Research and Development Fund, Grant‐in‐Aid for Scientific Research on Innovative Areas, and Hitachi Ltd.; grants and personal fees from Olympus; and personal fees from AstraZeneca, Novartis, COOK, AMCO Inc., Thermo Fisher Scientific, Erbe Elektromedizin GmbH, Fujifilm, Chugai, and Eli Lilly outside of the submitted work. Y.O. reports receiving grants from Roche and AbbVie K.K.; and personal fees from AstraZeneca, Ely Lilly K.K., Bristol‐Myers Squibb, Pfizer Taiho Pharma Co. Ltd., AstraZeneca Nippon Boehringer Ingelheim, Chugai Pharma Co. Ltd., Ono Pharma Co. Ltd., and Taiho Pharma Co. Ltd. outside of the submitted work. Y.G. reports receiving grants from AZK, AbbVie, Kyorin, and Preferred Network; grants and personal fees from Pfizer, Eli Lilly, Bristol‐Myers Squibb, Ono, Novartis, and Daiichi Sankyo; and personal fees from Chugai, Taiho, Boehringer Ingelheim, Merck Sharp & Dohme, Merck, Thermo Fisher, AstraZeneca, Chugai, Guardant Health Inc., and Illumina outside of the submitted work. H.H. reports receiving grants and personal fees from Merck Sharp & Dohme, AstraZeneca, Ono, Chugai, Roche, and Novartis; grants from AbbVie, Bristol‐Myers Squibb, Merck Biopharma, Daiichi Sankyo, Janssen, and Genomic Health; and personal fees from Eli Lilly and Kyowa‐Kirin, outside of the submitted work. N.Y. reports receiving grants from Chugai, Taiho, Eisai, Eli Lilly, Quintiles, Astellas, Bristol‐Myers Squibb, Novartis, Daiichi Sankyo, Pfizer, Boehringer Ingelheim, Kyowa‐Hakko Kirin, Bayer, Ono Pharmaceutical Co. Ltd., Takeda, Janssen Pharma, Merck Sharp & Dohme, Merck, GlaxoSmithKline, Sumitomo Dainippon, Chiome Bioscience Inc., Otsuka, Carna Biosciences, Genmab, and Shionogi; and personal fees from Ono Pharmaceutical Co. Ltd., Chugai, AstraZeneca, Pfizer, Lilly, Bristol‐Myers Squibb, Eisai, Otsuka, Takeda, Boehringer Ingelheim, Cimic, Sysmex, and Eisai, outside of the submitted work. Y.O. reports receiving grants, personal fees, and nonfinancial support from AstraZeneca, Chugai, Ono Pharmaceutical Co. Ltd., and Bristol‐Myers Squibb; grants and personal fees from Eli Lilly and Pfizer; grants and nonfinancial support from Kyorin; grants from Dainippon‐Sumitomo, Taiho, Novartis, Takeda, Kissei, Daiichi Sankyo, Janssen, and LOXO; and personal fees from Boehringer Ingelheim, Bayer, Merck Sharp & Dohme, Taiho, Nippon Kayaku, Kyowa‐Hakko Kirin, Celltrion, Amgen, and AnHeeart Therapeutics Inc. outside of the submitted work. The other authors declare no conflicts of interest.

## Supporting information


**Figure S1.** Patient selection
**Figure S2**. Kaplan–Meier curves for (A) DFS and (B) OS in patients with limited‐stage small cell lung cancer who received adjuvant chemotherapy after surgery. DFS, disease‐free survival; OS, overall survival.

## Data Availability

The datasets generated during the current study are not publicly available owing to ethical restrictions but are available from the corresponding author upon reasonable request.
