# Supplementary material for: Schlafen 11 Expression in Patients With Small Cell Lung Cancer and Its Association With Clinical Outcomes
Source: Thorac Cancer. 2025 Jan 14;16(1):e15529. doi: 10.1111/1759-7714.15529 (PMC11732703; doi:10.1111/1759-7714.15529)
Supplement: Supplementary file 1 — Figure S1. Patient selection Figure S2. Kaplan–Meier curves for (A) DFS and (B) OS in patients with limited‐stage small cell lung cancer who received adjuvant chemotherapy after surgery. DFS, disease‐free survival; OS, overall survival. [file TCA-16-e15529-s001.pptx]

## Slide 1
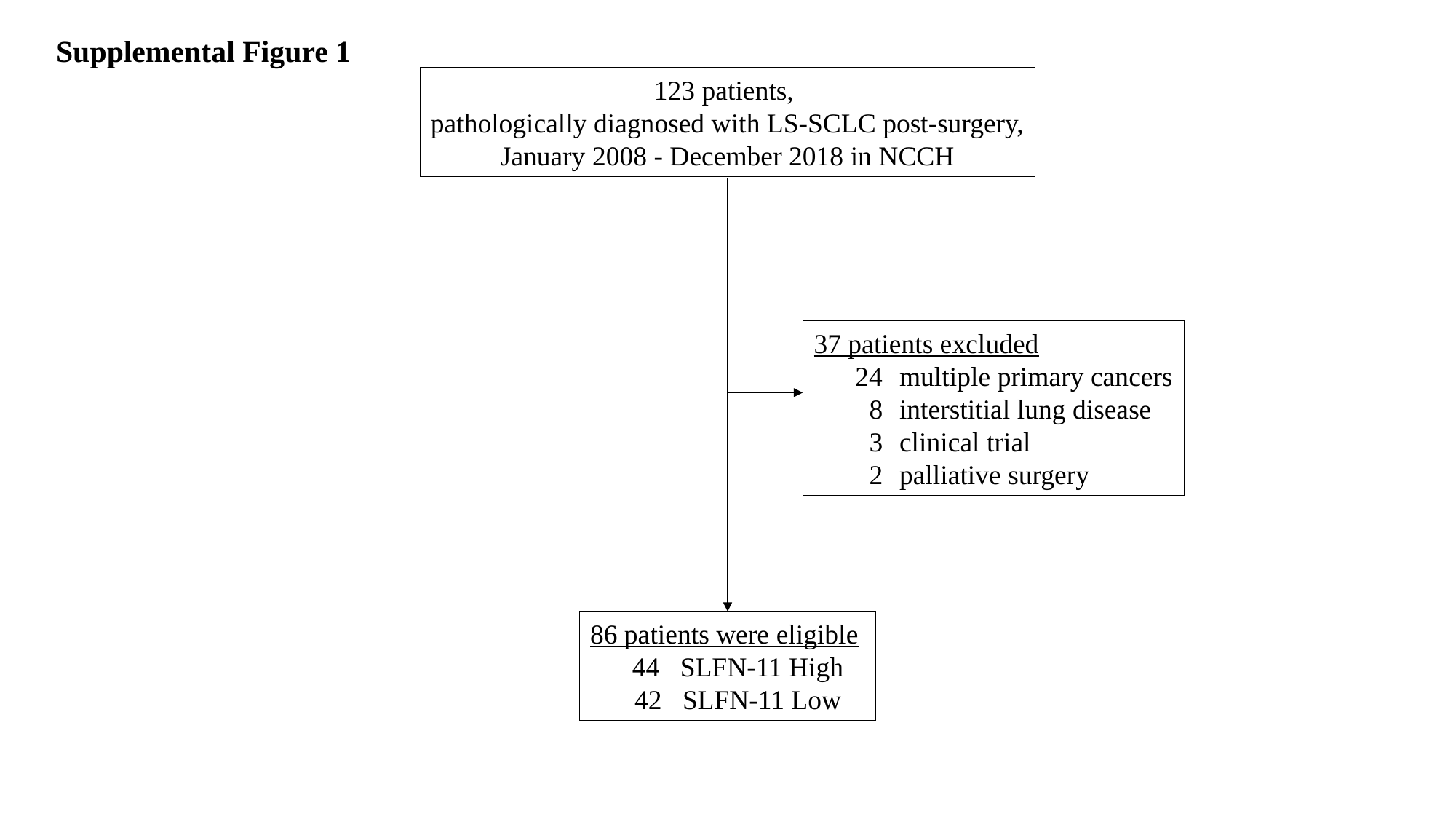

Supplemental Figure 1
123 patients,
pathologically diagnosed with LS-SCLC post-surgery,
January 2008 - December 2018 in NCCH
37 patients excluded
 24	multiple primary cancers
 8 	interstitial lung disease
 3	clinical trial
 2	palliative surgery
86 patients were eligible
 44 SLFN-11 High
 42 SLFN-11 Low

## Slide 2
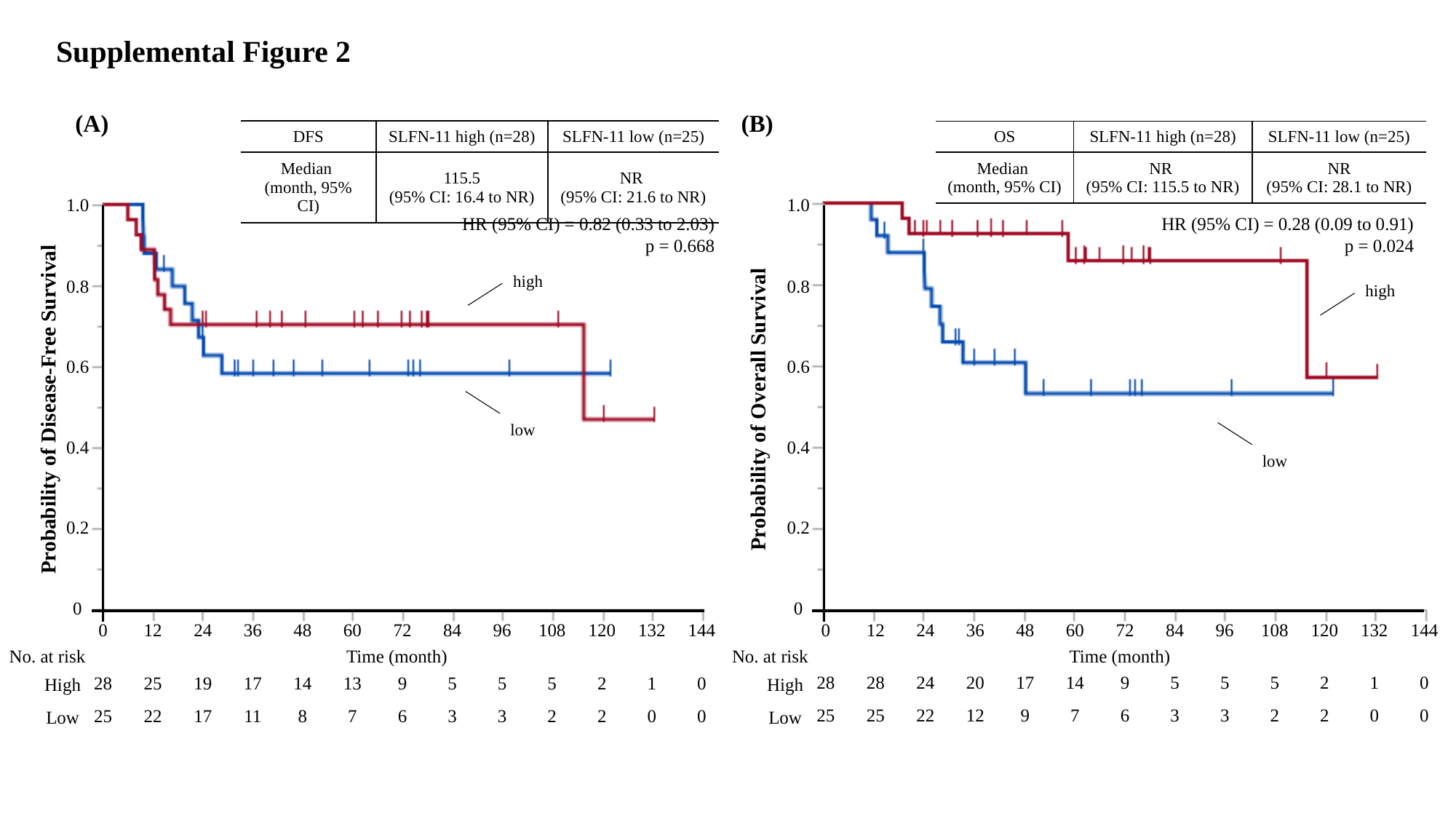

Supplemental Figure 2
(B)
(A)
| DFS | SLFN-11 high (n=28) | SLFN-11 low (n=25) |
| --- | --- | --- |
| Median (month, 95% CI) | 115.5 (95% CI: 16.4 to NR) | NR (95% CI: 21.6 to NR) |
| OS | SLFN-11 high (n=28) | SLFN-11 low (n=25) |
| --- | --- | --- |
| Median (month, 95% CI) | NR (95% CI: 115.5 to NR) | NR (95% CI: 28.1 to NR) |
| 1.0 |
| --- |
| 0.8 |
| 0.6 |
| 0.4 |
| 0.2 |
| 0 |
| 1.0 |
| --- |
| 0.8 |
| 0.6 |
| 0.4 |
| 0.2 |
| 0 |
HR (95% CI) = 0.82 (0.33 to 2.03)
p = 0.668
HR (95% CI) = 0.28 (0.09 to 0.91)
p = 0.024
high
high
Probability of Disease-Free Survival
Probability of Overall Survival
low
low
| 0 | 12 | 24 | 36 | 48 | 60 | 72 | 84 | 96 | 108 | 120 | 132 | 144 |
| --- | --- | --- | --- | --- | --- | --- | --- | --- | --- | --- | --- | --- |
| 0 | 12 | 24 | 36 | 48 | 60 | 72 | 84 | 96 | 108 | 120 | 132 | 144 |
| --- | --- | --- | --- | --- | --- | --- | --- | --- | --- | --- | --- | --- |
No. at risk
Time (month)
No. at risk
Time (month)
High
| 28 | 28 | 24 | 20 | 17 | 14 | 9 | 5 | 5 | 5 | 2 | 1 | 0 |
| --- | --- | --- | --- | --- | --- | --- | --- | --- | --- | --- | --- | --- |
| 25 | 25 | 22 | 12 | 9 | 7 | 6 | 3 | 3 | 2 | 2 | 0 | 0 |
High
| 28 | 25 | 19 | 17 | 14 | 13 | 9 | 5 | 5 | 5 | 2 | 1 | 0 |
| --- | --- | --- | --- | --- | --- | --- | --- | --- | --- | --- | --- | --- |
| 25 | 22 | 17 | 11 | 8 | 7 | 6 | 3 | 3 | 2 | 2 | 0 | 0 |
Low
Low
